# Left Ventricular Non-compaction Cardiomyopathy: A Report of a Rare Case From Saudi Arabia

**DOI:** 10.7759/cureus.64937

**Published:** 2024-07-19

**Authors:** Husna Irfan Thalib, Sayeeda Mehveen, Sariya Khan, Shyma Haidar, Ayesha Jamal, Ayesha Shaikh, Mohammed A Alfaqih, Amir A Mansy

**Affiliations:** 1 General Medicine and Surgery, Batterjee Medical College, Jeddah, SAU; 2 Genetics, St. Ann's College for Women, Hyderabad, IND; 3 Internal Medicine, Faculty of Medicine, King Abdulaziz University, Jeddah, SAU; 4 Cardiology, King Abdullah Medical Complex - Jeddah, Jeddah, SAU

**Keywords:** guideline-directed medical therapy, spongy myocardial tissue, trabeculations, echocardiogram, rare congenital heart disease, left ventricular noncompaction cardiomyopathy

## Abstract

Left ventricular non-compaction cardiomyopathy (LVNC) is an unusual congenital heart disease that predominantly affects the heart’s left ventricle. This disease is characterized by deep intertrabecular recesses and hypertrabeculations of the myocardial wall that link with the ventricle cavity. During embryogenesis, the fetal myocardium has to undergo a compaction process, wherein the trabeculated and spongy myocardial tissue compacts into a dense, solid form. An incomplete compaction process results in persistent non-compacted spongy myocardial tissue and trabeculations prominent in the left ventricle. This disease could be marked alone or be present in coexistence with other congenital heart abnormalities. We present a rare case of a 57-year-old Saudi male who presented to the ER with chest pain and dyspnea. Due to severe chest pain, he was admitted to the coronary care unit. On further investigation, an echocardiogram revealed heavy trabeculations in the dilated left ventricle and a reduced ejection fraction. The case was diagnosed as LVNC and possible heart failure. The patient was discharged after he was kept under guideline-directed medical therapy (GDMT) along with certain medications and will be evaluated after six months of GDMT to decide on implantable cardiac resynchronization therapy. Although LVNC is rare, it can lead to severe heart conditions like arrhythmias, thromboembolism, and heart failure.

## Introduction

The most recently classified form of cardiomyopathy, left ventricular non-compaction cardiomyopathy (LVNC), presents with irregular trabeculations in the left ventricle, which are most frequent and prominent at the apex [1]. It can be accompanied by left ventricular hypertrophy, and systolic or diastolic dysfunction, with or without right ventricular involvement. Most patients are asymptomatic; however, symptoms of heart failure (HF) may present during exercise [2]. The life-threatening risks of LVNC include complete atrioventricular block or ventricular arrhythmias that can present as syncope or sudden death [3]. There is a risk of thromboembolic events. However, given the available data, it is not clear whether the thromboembolic rate in patients with LVNC differs from the rate generally observed in patients with HF. Metabolic abnormalities and disrupted mitochondrial function may be causes of LVNC development. Furthermore, it was found that certain genes may also have a role in this disease's development. Genetic inheritance was found in 30-50% of patients [1]. Diagnosing LVNC is complex because there are diverse ways to define it. A diagnostic criteria, introduced by Chin et al., measures the ratio between the thickness of the heart muscle and the depth of its inner recesses. Another method, proposed by Jenni et al., examines the thickness ratio between different layers of the heart muscle. Additionally, Stollberger et al. suggest counting visible heart wall projections in certain views. There's an ongoing debate about which imaging method is best for confirming LVNC, including angiography, echocardiography, computed tomography, and magnetic resonance imaging (MRI). Echocardiography and MRI are often preferred because it provides detailed images of heart tissue. Ultimately, diagnosing LVNC involves considering different criteria and choosing the most suitable imaging technique based on the patient's condition [2]. Treatment aims to enhance heart function and alleviate strain on the heart in individuals with systolic dysfunction. Managing arrhythmias and, when appropriate, implanting a cardioverter-defibrillator to prevent sudden cardiac death are crucial aspects of therapy. For those with both LVNC and congenital heart issues, surgical or catheter-based interventions may be necessary. Despite advancements in diagnosis and treatment over the last decade, there's still much to learn about this condition and how best to improve patient outcomes [4]. In this case report, we discuss a 57-year-old man who presented to the clinic with a chief complaint of dyspnea and chest pain and was later diagnosed with LVNC.

## Case presentation

**Figure 1:**
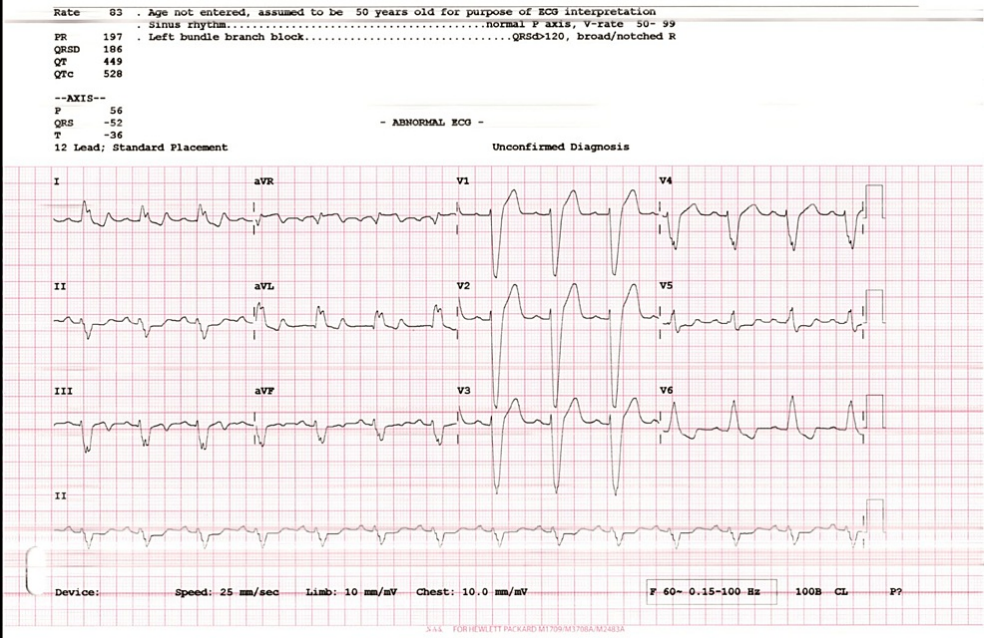
ECG demonstrates normal sinus rhythm with left bundle branch block.

A 57-year-old man presented to the ER with a chief complaint of dyspnea and chest pain. His past medical history revealed that he is a regular smoker and is currently on two home medications: aspirin and statin. There were no significant findings in his family history or allergies. His past surgical history revealed that he underwent coronary angiography (CAG) 24 years ago and was told that he had no significant lesions. The patient was admitted to the coronary care unit (CCU) with unstable angina. During the clinical exam, his blood pressure (BP) was recorded to be 124/80 mmHg, congested neck veins, his heart rate was 50 bpm, S1 and S2 were heard with no added sounds or murmurs. Chest reveals fine basal crackles and lower limbs (LL) reveal no edema and lax calf muscles. His ECG as demonstrated in Figure 1 revealed a normal sinus rhythm with a left bundle branch block.

**Figure 2:**
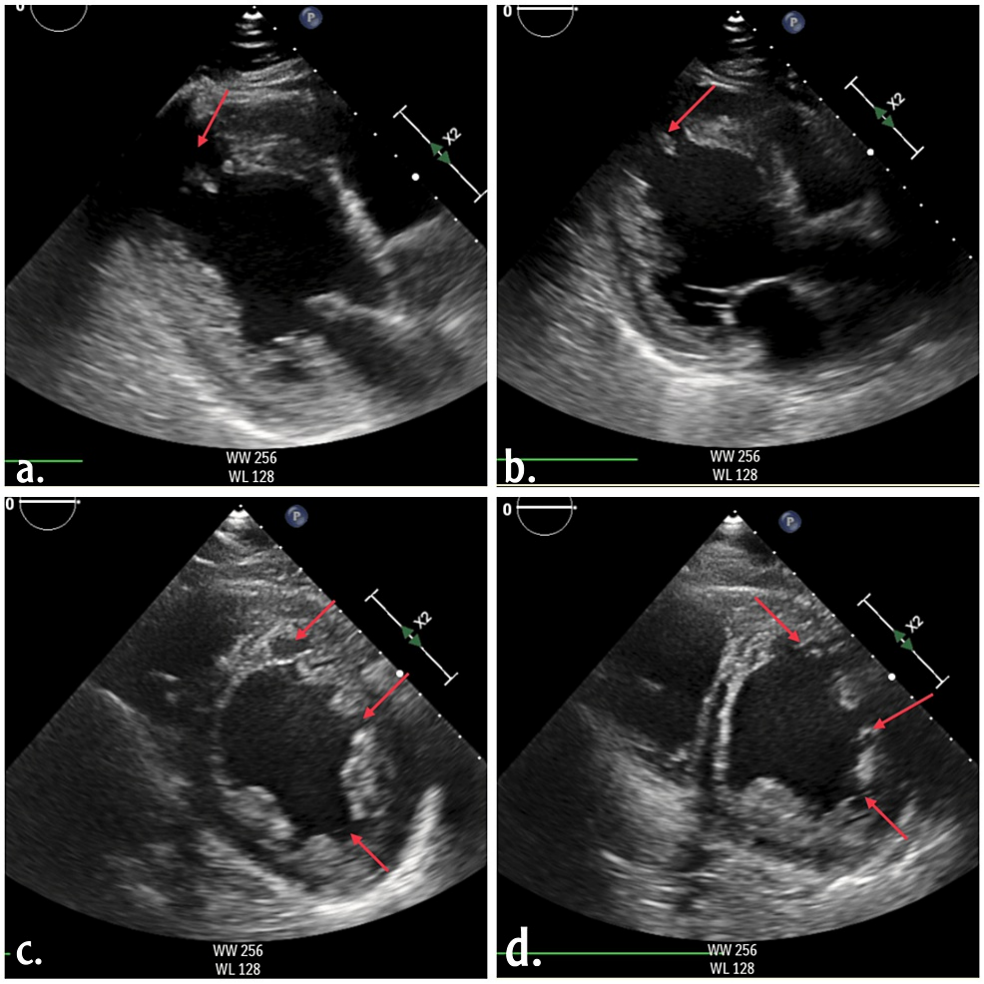
Echocardiogram (a-d) indicates dilated left ventricle internal dimensions, heavy trabeculations, global hypokinesia, ejection fraction was 20-25% by 2D eyeballing, the right side was normal in size with normal right ventricular function, no intracardiac masses or thrombi, normal pericardium with no pericardial effusion.

The echocardiogram as attached in Figure 2 revealed significant left ventricular dysfunction with an ejection fraction of 20-25%. Extensive trabeculations were observed in the apex, apical, and basal segments. Initially suspected as left ventricular thrombi, further imaging clarified these as thickened myocardial walls with pronounced trabeculations and deep recesses. Further lab investigations as mentioned in Table 1 revealed a negative test for troponin, normal blood count and normal renal functions, normal HBA1c and normal lipid profile. These findings suggested a diagnosis of HF with reduced ejection fraction (HFrEF) and possible non-compaction cardiomyopathy.

**Table 1:** Laboratory findings Lab investigations revealed a negative test for troponin, normal blood count, normal renal functions, normal HBA1c and a normal lipid profile.

Test	Result	Reference Range
Chloride - Plasma or Serum	103	98-107 mmol/L
Creatinine	100	62.0-115.0 umol/L
Potassium	4.2	3.5-5.1 mmol/L
Sodium - Plasma or Serum	139	136-145 mmol/L
Blood Urea Nitrogen (BUN)	6.4	2.5-8.0 mmol/L
Turbo Troponin I	0.02	0.020-0.060 ug/L
Prothrombin Time (PT)	15.5	11.50-14.50 sec
International Normalized Ratio (INR)	1.2	0.8-1.1
Activated Partial Thromboplastin Time (APTT)	45.3	22.5-37.5 sec
Cholesterol - Total	4.5	0.0-5.2 mmol/L
Cholesterol (HDL)	1	1-2 mmol/L
Cholesterol (LDL)	2	<3 mmol/L
Triglycerides	3.88	<1.70 mmol/L
Glycosylated Hemoglobin	5.4	4.0-5.6%

**Figure 3:**
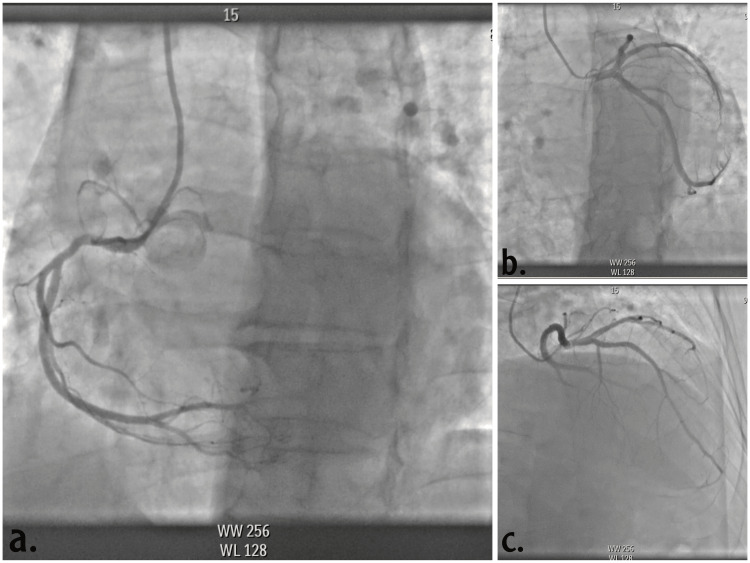
The coronary angiography (a, b, c) revealed normal coronaries.

CAG revealed normal coronaries as demonstrated in Figure 3. The patient was kept on guideline-directed medical therapy (GDMT) including sacubitril/valsartan 50 bid, carvedilol 25 mg bid, spironolactone 25 OD and empagliflozin 10 mg OD, furosemide 40 mg OD, we started also apixaban 5 mg bid as oral anticoagulation. The patient was discharged with subsequent follow-up and cardiac MRI in the OPD.

**Figure 4:**
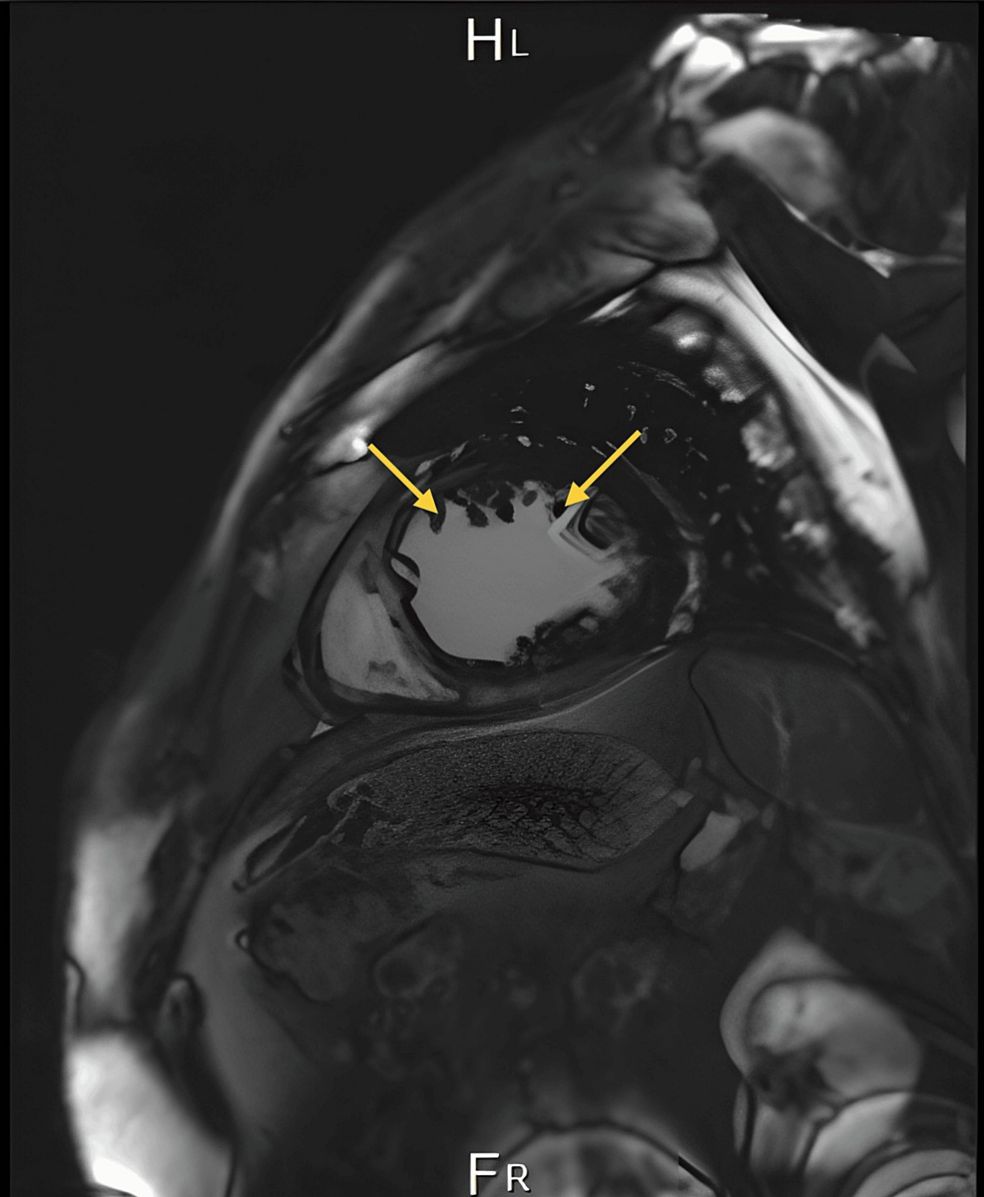
MRI demonstrated typical morphology of left ventricular non-compaction cardiomyopathy, prominent in the left ventricle endocardium with more than 3 villi below the papillary muscle (view 1).

**Figure 5:**
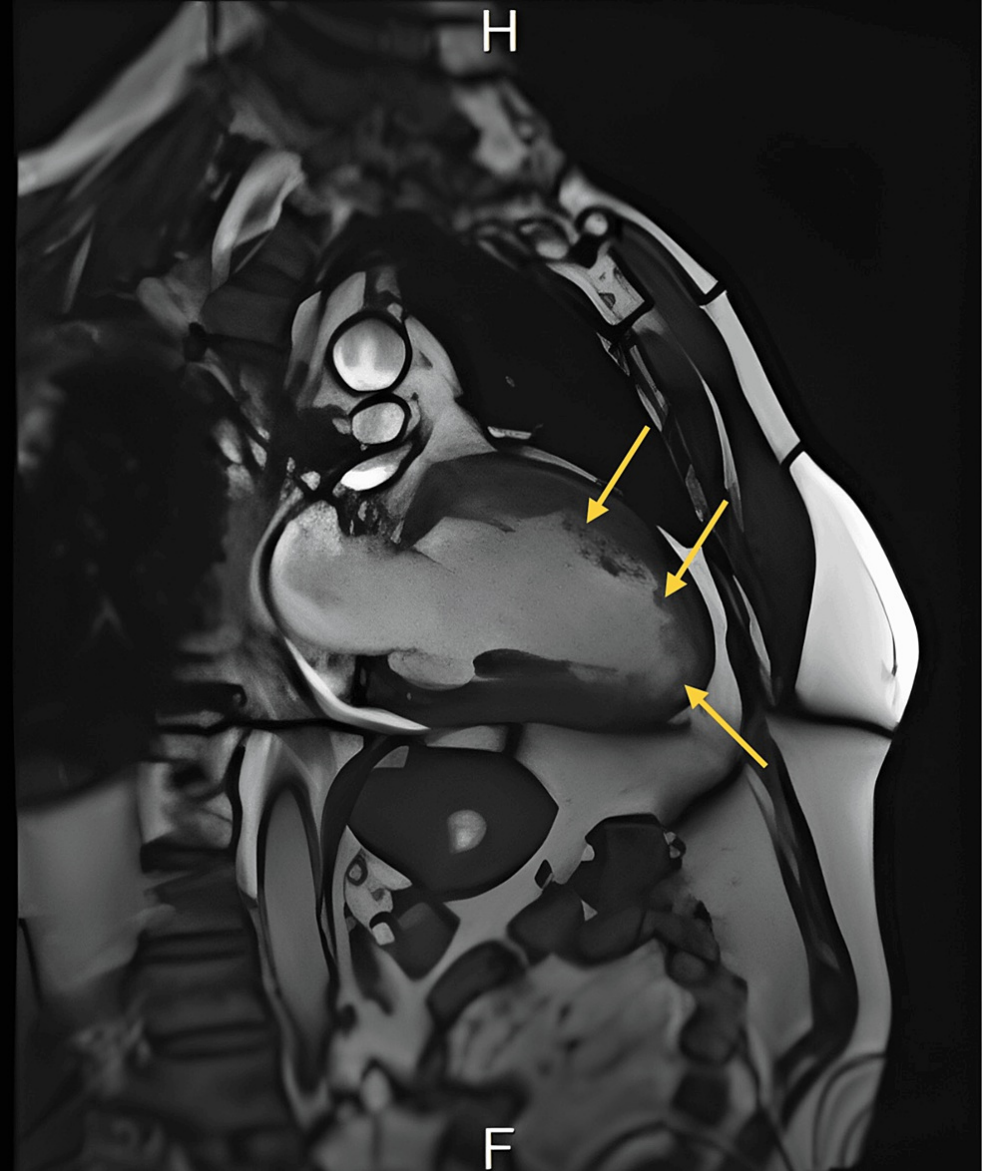
MRI demonstrated typical morphology of left ventricular non-compaction cardiomyopathy, prominent in the left ventricle endocardium with more than 3 villi below the papillary muscle (view 2).

Cardiac MRI as attached in Figures 4-5 demonstrated typical morphology of LVNC, prominent in the left ventricle endocardium with more than 3 villi below the papillary muscle. MRI confirmed the diagnosis. The left ventricular non-compaction to compacted myocardium ratio was esteemed to be more than 40% of the left ventricle.

The long-term follow-up plan includes GDMT and non-vitamin K antagonist (non-VKA) oral anticoagulants as prophylaxis measures. After six months of GDMT, the patient’s condition will be further assessed to decide on an implantable cardiac resynchronization therapy (CRT).

## Discussion

The represented case of a 57-year-old man indicates non-compaction cardiomyopathy, a rare condition affecting 8-12 individuals per one million per year occurring mostly in men compared to women. This is an extremely rare occurrence in Saudi Arabia with few reported cases [5]. Non-compaction cardiomyopathy develops due to the failure of myocardial fibres to compact during the embryonic stage. This results in a thin epicardial layer, and a spongier endocardial layer caused by trabeculations, hence making the muscle in the left ventricle less firm and smooth. This leads to dysfunction of normal heart functions, resulting in symptoms of HF, arrhythmias, and sometimes presenting as severe chest pain (angina), as revealed in our case [6].

The diagnosis of non-compaction cardiomyopathy is often indistinguishable and can be confusing when the patient has left ventricular hypertrophy; it requires proper clinical evaluation followed by radiological imaging, with echocardiography being the most important [7]. The Jenni criteria are the most accepted and widely used criteria for diagnosis by echocardiography, although some clinicians may use the Chin or Stöllberger criteria [8]. As per the Jenni criteria for LVNC diagnosis, assessment must be done in the parasternal short-axis view at the base, mid, and apical levels. All four echocardiographic criteria must be present: (1) A thickened LV wall with a thin compacted epicardial layer and a thick endocardial layer with many trabeculations and deep recesses, with a non-compacted to compacted myocardium ratio >2:1 at end-systole; (2) Color Doppler showing blood flow within the deep intertrabecular recesses; (3) Prominent trabecular meshwork in the LV apex or midventricular segments of the inferior and lateral wall, possibly affecting the right ventricle; (4) Compacted wall thickness ≤8.1 mm, indicating myocardial thickening specific to LVNC [9]. In our patient, the echocardiography results revealed a dilated left ventricle (LV), global hypokinesia with heavy LV trabeculations indicative of HFrEF, and possible non-compaction cardiomyopathy.

Cardiac magnetic resonance (CMR) plays a crucial role in the diagnosis of LVNC when echocardiographic findings are inconclusive. Unlike echocardiography, CMR provides detailed morphologic information, including the ability to detect fibrosis through late gadolinium enhancement (LGE), which can offer valuable prognostic insights. Typically, CMR is performed initially to confirm LVNC diagnosis and is repeated as necessary during follow-up to assess evolving clinical symptoms and complications [9].

In CMR assessment for LVNC, specific criteria such as the X/Y ratio are applied, where X represents the distance from the epicardial surface to the trough of trabecular recesses and Y is from the epicardial surface to the peak of trabeculations. This measurement is crucial in the subxiphoid or apical four-chamber views during end-diastole. However, determining the sensitivity and specificity of CMR remains challenging due to the absence of a definitive gold standard. Key characteristics of LVNC on CMR include the presence of a non-compacted myocardial layer in the left ventricle, which CMR can visualize with high spatial resolution across all LV segments, including the apex and lateral wall. Quantitative criteria play a critical role in distinguishing LVNC from other conditions, as regions showing apparent non-compaction can be found in both LVNC and other groups with or without cardiovascular disease. Proposed CMR criteria for LVNC diagnosis include measurements such as a maximum end-diastolic non-compacted to compacted myocardial thickness ratio >2.3, which has demonstrated high sensitivity (86%) and specificity (99%) in differentiating LVNC from controls in various studies. Additionally, a trabeculated LV mass exceeding 20% of the global LV mass has shown promising sensitivity and specificity rates of 94%. The fractal dimension, another quantitative measure of trabeculations, is noted for its high accuracy and reproducibility, with variations observed particularly in the apical third of the LV between different demographic groups [9].

Some patients with this condition show no symptoms and maintain normal left ventricular (LV) systolic function, while others develop HF, thromboembolic events, and severe arrhythmias. Systolic dysfunction and arrhythmias may result from dysfunction at the microcirculation level and reduced blood flow to the subepicardial region. The management of non-compaction cardiomyopathy is the same for HF cases with some considerations [10].

In our case, the patient was vitally stable and complaining of dyspnea in New York Heart Association (NYHA) class II-III; he was discharged on GDMT for HF. The medications included the four pillars for HF (sacubitril/valsartan, spironolactone, empagliflozin, and carvedilol) and also he was discharged on apixaban 5 mg bid for the risk of thromboembolism. The patient was requested to follow up and undergo an MRI. The results of the MRI confirmed LVNC with a prominent left ventricular endocardium with more than 3 villi below the papillary muscle. The left ventricular non-compaction to compacted myocardium ratio was estimated to be more than 40% of the left ventricle. The patient was then discharged on the same medications to the maximum tolerated dose of GDMT. After six months, he is supposed to be evaluated for possible implantation of a cardiac resynchronization therapy with a defibrillator (CRT-D) since his ECG showed a left bundle branch block (LBBB) with QRS complex 160 milliseconds. Some patients have poor prognosis, and the management can be escalated to the level of left ventricular assisted device (LVAD) or heart transplantation.

Our 57-year-old Saudi male patient presented with dyspnea and chest pain and revealed classic echocardiographic and MRI features of LVNC. Similarly, a 62-year-old Saudi woman demonstrated progressive dyspnea and chest pain, ultimately diagnosed with LVNC and myocardial bridging (MB). Despite a lack of risk factors, her management included diuretics and conventional HF therapy to manage symptoms and thromboembolic risk [11]. A similar case by Okan et al. in 2023 reported a 62-year-old male with LVNC presenting for arrhythmia evaluation, managed with beta-blockers, angiotensin-converting enzyme (ACE) inhibitors, and later sacubitril/valsartan, showing improvement in left ventricular ejection fraction (LVEF) over five years of follow-up [12]. Huang et al. in 2022 discussed a 61-year-old female with LVNC and significant right ventricular involvement, managed with diuretics, sacubitril/valsartan, and antiarrhythmics, emphasizing the role of comprehensive management in complex cases [13]. Rani et al. in 2020 reported a 65-year-old male with LVNC presenting with stroke-like symptoms, managed with diuretics, sacubitril/valsartan, and anticoagulation for secondary stroke prevention, emphasizing the importance of holistic care [14].

According to our literature search, there are few cases of LVNC reported in Saudi Arabia. This case highlights the importance of making an accurate diagnosis, as it can mimic other forms of cardiomyopathy, and increase awareness among healthcare professionals. It also emphasizes the importance of early diagnosis for appropriate management and improved outcomes. This case report has provided valuable insights; however, it has limitations as this report is based on a single patient's experience, and it does not account for diverse outcomes or underlying pathophysiology. Determining an optimal management plan requires a larger sample size.

## Conclusions

The presented case of a 57-year-old with LVNC indicates the rarity of this condition in Saudi Arabia and highlights the challenges in its diagnosis and management. Through careful clinical evaluation and radiological imaging, a definitive diagnosis was achieved, emphasizing the importance of awareness among healthcare professionals regarding this often-overlooked condition. The management strategy in this case, consisted of GDMT, similar to the plan mentioned in other cases previously discussed, followed by subsequent consideration of CRT-D implantation, demonstrating the tailored approach necessary for addressing the varying presentations and severity of symptoms in patients with non-compaction cardiomyopathy. While this report contributes valuable insights to the medical literature, it also points out the need for further research to explain the underlying pathophysiology, optimize diagnostic criteria, and refine therapeutic strategies. Furthermore, continuous monitoring and symptom assessment are crucial because early detection and intervention play a key role in managing the progression of this complex disease and enhancing patient outcomes.
